# Interpretable machine learning for early predicting the risk of ventilator-associated pneumonia in ischemic stroke patients in the intensive care unit

**DOI:** 10.3389/fneur.2025.1513732

**Published:** 2025-05-07

**Authors:** Heshan Cao, Junying Wei, Ping Hua, Songran Yang

**Affiliations:** ^1^Department of Neurology, Sun Yat-sen Memorial Hospital, Sun Yat-sen University, Guangzhou, China; ^2^Department of Anaesthesiology, The First Affiliated Hospital of Guangzhou University of Chinese Medicine, Guangzhou, China; ^3^Department of Cardio-Vascular Surgery, Sun Yat-sen Memorial Hospital, Sun Yat-sen University, Guangzhou, China; ^4^Department of Biobank and Bioinformatics, Sun Yat-sen Memorial Hospital, Sun Yat-sen University, Guangzhou, China

**Keywords:** ischemic stroke, ventilator-associated pneumonia, machine learning, SHAP, MIMIC-IV database

## Abstract

**Background:**

The incidence of ventilator-associated pneumonia (VAP) in ischemic stroke (IS) patients is linked to a variety of detrimental outcomes. Current approaches for the early identification of individuals at high risk for developing VAP are limited and often lack clinical interpretability. The goal of this study is to develop and validate an interpretable machine learning (ML) model for early predicting VAP risk in IS patients in the intensive care unit (ICU).

**Methods:**

Data on IS patients were extracted from versions 2.2 and 3.0 of the Medical Information Mart for Intensive Care-IV database, with version 2.2 being used for model training and internal validation and version 3.0 for external testing. The primary outcome was the incidence of VAP post-ICU admission. The Boruta algorithm was used to select features prior to developing 10 ML models. The Shapley Additive Explanation (SHAP) method was employed to assess the global and local interpretability of the model’s decision-making process. The final model and Streamlit were used for developing and launching an online web application.

**Results:**

A total of 419 IS patients were included, with 401 in the derivation and 118 in the test group. Following feature selection, seven clinical characteristics were incorporated in the ML model: systolic and diastolic blood pressure, international normalized ratio, length of stay before mechanical ventilation, dysphagia, antibiotic counts and suctioning counts. Among the 10 evaluated ML models, the Random Forest (RF) model outperformed the others, achieving an internal validation AUC of 0.776, accuracy of 0.704, sensitivity of 0.900, and specificity of 0.588. In external testing, performance dropped to an AUC of 0.644, accuracy of 0.610, sensitivity of 0.688, and specificity of 0.519, raising concerns about the model’s generalizability.

**Conclusion:**

The RF model is reliable in early identifying high-risk IS patients for VAP. The SHAP method offers clear and intuitive explanations for individual risk assessment. The web-based tool has the potential to improve clinical outcomes by promptly recognizing patients at increased VAP risk and facilitating early intervention, further multicenter prospective studies are required to validate its generalizability and practical utility.

## 1 Introduction

According to World Stroke Organization statistics for 2022, stroke continues to be the second leading cause of mortality and the third leading cause of disability worldwide, thereby posing a significant threat to public health (1). Ischemic stroke (IS) is the most prevalent type, accounting for 60%–70% of all instances (2). Mechanical ventilation (MV) is frequently essential to prevent potentially fatal respiratory failure or apnea in IS patients, particularly in the intensive care unit (ICU), due to the significant neurological abnormalities these patients frequently suffer.

Stroke patients undergoing MV are at increased risk for a serious pulmonary complication known as ventilator-associated pneumonia (VAP), which may have a devastating impact on their respiratory function and overall prognosis (3). Early and accurate identification of IS patients at high risk for VAP remains a critical yet challenging aspect of clinical management, as delayed diagnosis can result in worsened patient outcomes and increased clinical burden (4, 5).

Although machine learning (ML) methods have demonstrated promising results in predictive modeling within clinical research (6, 7), early predictive models specifically targeting VAP risk in IS patients remain scarce. To address this gap, we developed and validated an interpretable ML model utilizing stroke-related clinical data from a large public database. The SHapley Additive exPlanation (SHAP) method (8) was employed to enhance the interpretability of predictions. We also constructed an accessible web-based tool designed to assist clinicians in rapidly identifying IS patients at increased VAP risk.

## 2 Methods

### 2.1 Study population

The Medical Information Mart for Intensive Care (MIMIC)—IV database, specifically versions 2.2 and 3.0, was used for this retrospective analysis (9, 10). MIMIC-IV is a publicly accessible, large-scale intensive care database organized and maintained by the Laboratory for Computational Physiology at the Massachusetts Institute of Technology (MIT). This study utilized version 2.2, which includes medical data from approximately 300,000 patients treated at the Beth Israel Deaconess Medical Center (BIDMC) from 2008 to 2019, for model training and internal validation. Version 3.0, which includes data from 2020 to 2022 was utilized for model external testing. The use of MIMIC-IV data was ethically approved by the Institutional Review Boards of BIDMC and MIT. Since all personal data in the database are anonymized, informed consent was waived. The author (Heshan Cao) was granted access to the database (certification number: 63137030). Reporting of this study followed the Transparent Reporting of a Multivariable Prediction Model for Individual Prognosis or Diagnosis (TRIPOD+AI) guidelines (Supplementary Table 1) (11).

**Figure 1:**
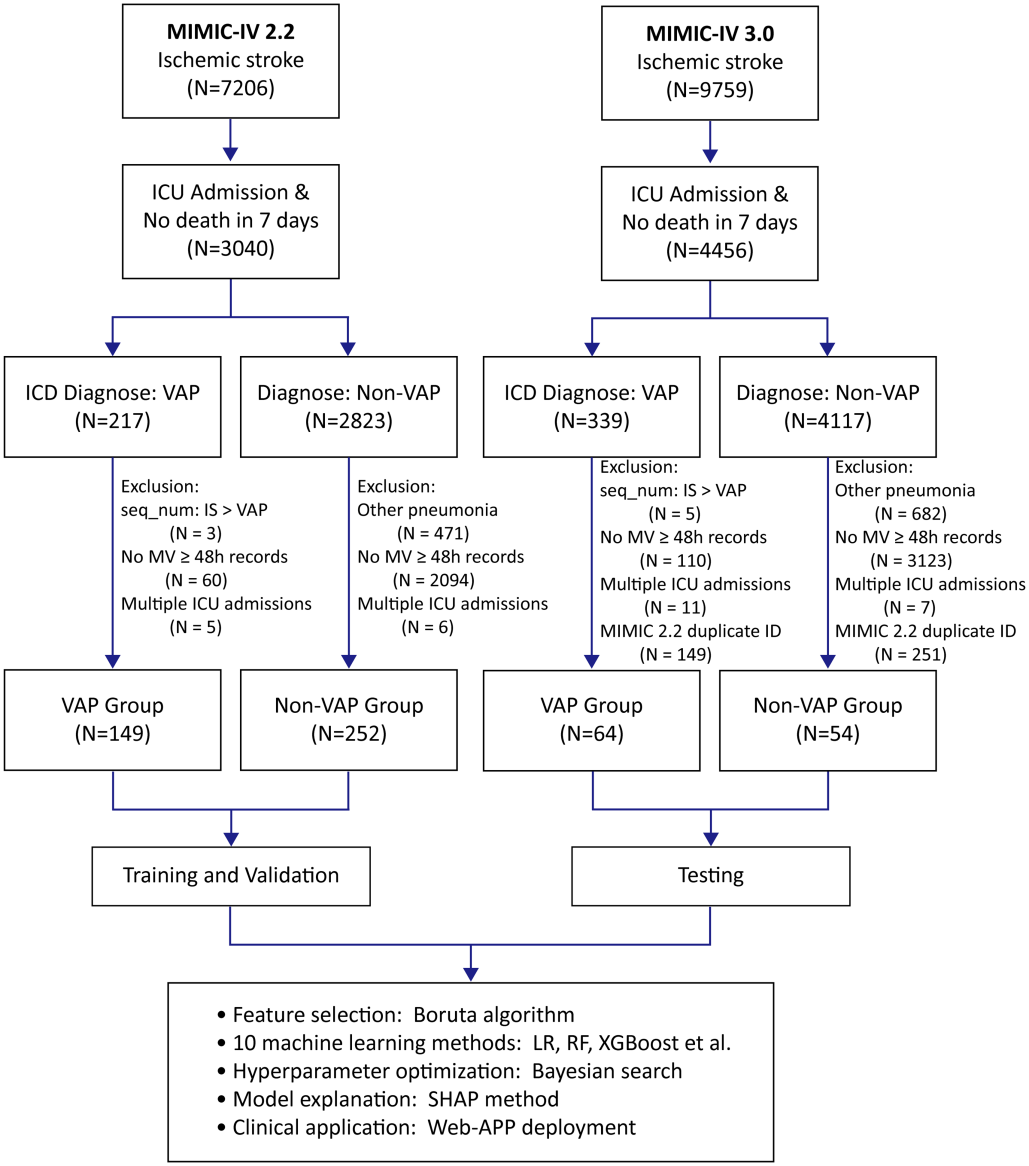
Inclusion and exclusion flowchart and study workflow.

This study included patients aged 18 and above who were admitted to the ICU primarily for IS and had MV for more than 48 h. The primary outcome measure was the incidence of VAP. Patients with IS and VAP were identified in the MIMIC-IV database using the International Classification of Diseases, Ninth Revision (ICD-9), or Tenth Revision (ICD-10) codes. By restricting the diagnostic sequence to IS > VAP, we ensured that the diagnosis of IS was prioritized over that of VAP. Supplementary Table 2 lists the relevant ICD codes. Exclusion criteria included VAP diagnoses preceded IS and deaths within 7 days of ICU admission. For patients who had multiple ICU admissions, only the initial admission was considered. Figure 1 depicts a flowchart of inclusion and exclusion criteria.

### 2.2 Data collection and feature selection

The MIMIC-IV database was queried using Structured Query Language (SQL) to extract features such as demographics, comorbidities, vital signs, laboratory test indicators, and ventilator settings. Records within the first 24 h of MV were used to extract data on vital signs, laboratory indicators, and ventilator settings; variables with multiple records were averaged. Records were also acquired for antibiotic usage, suctioning procedures, and invasive catheter placements during the first 24 h of MV. We encoded comorbidities and VAP incidence as binary values.

**Figure 2:**
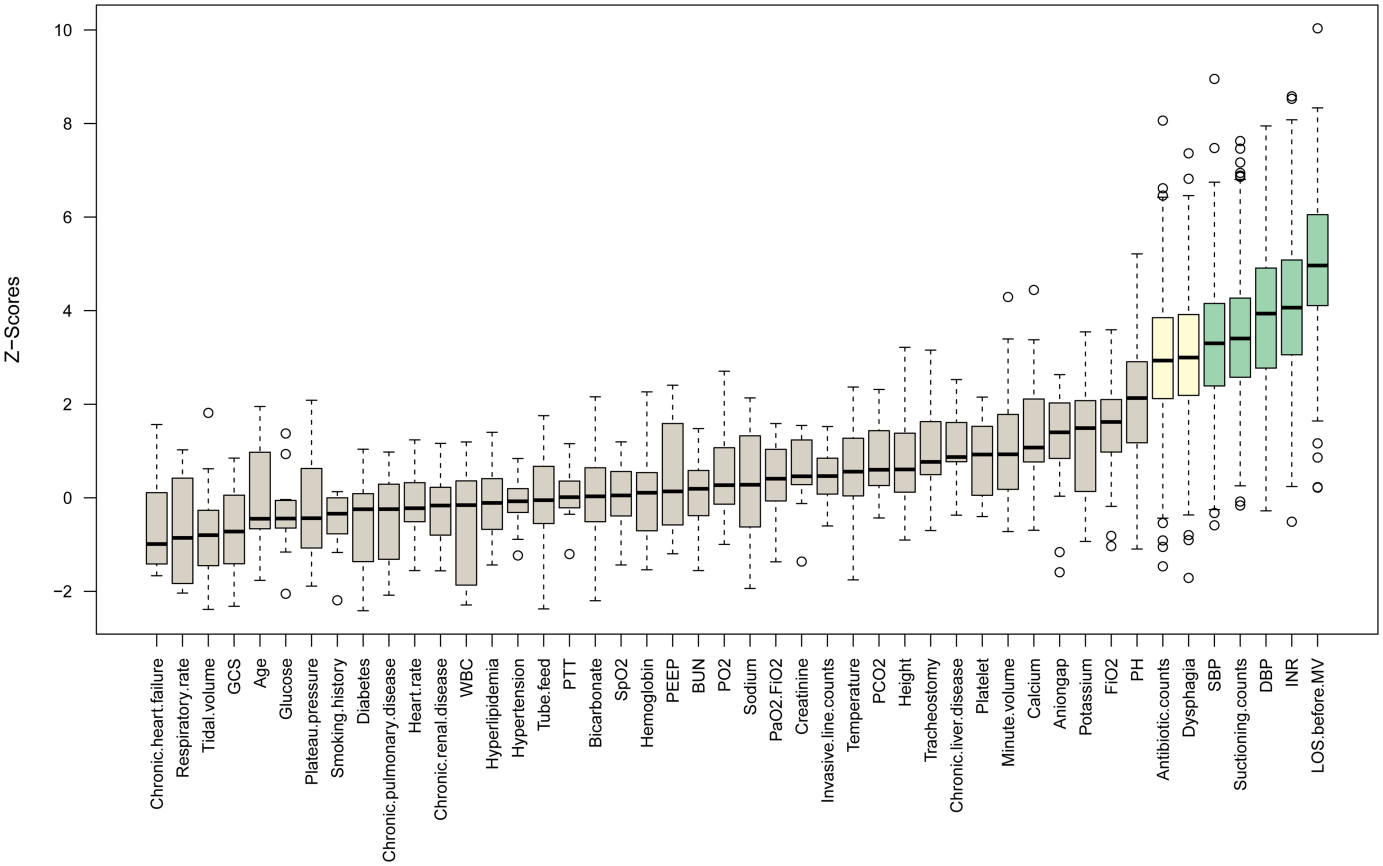
Results of feature selection using the Boruta algorithm.

A total of 59 features were obtained. Features with more than 20% missing data were first excluded to reduce missing data bias. Features with a missing data rate below 20% were addressed using multiple imputation methods, as indicated in Supplementary Figure 1. Following that, a correlation analysis was conducted on all features, and those with a correlation coefficient greater than 0.7 were excluded to prevent multicollinearity from impacting model performance (Supplementary Figure 2). Finally, the Boruta algorithm was applied to select the most relevant features. Boruta is an all-relevant feature selection method that uses a random forest (RF) classifier to compare the importance of original features with that of randomly permuted “shadow” features. By iteratively eliminating features that do not outperform their shadow counterparts, the algorithm robustly identifies truly informative features (12). In this study, Boruta was executed with a confidence level of 0.01, iterated 500 times to exclude rejected features, as presented in Figure 2.

### 2.3 Model development

The MIMIC-IV database data, which ranges from 2008 to 2019, was randomly divided into two sets: 80% for training and 20% for validation, using a stratified sampling strategy. To predict the risk of VAP in IS patients, 10 widely recognized ML models based on different principles were constructed: adaptive boosting (AdaBoost), category boosting (CatBoost), extra trees (ET), light gradient boosting machine (LightGBM), logistic regression (LR), multilayer perceptron (MLP), naive Bayes (NB), RF, support vector machine (SVM), and extreme gradient boosting (XGBoost). This comprehensive approach allowed us to identify the model that best balances performance for early VAP prediction in IS patients. To optimize the prediction models and avoid overfitting, the final hyperparameters for each model were determined using a combination of five-fold cross-validation and Bayesian search. For external validation, the trained models were tested on the MIMIC-IV database version 3.0, which covers the years 2020–2022.

The models’ performance was evaluated using metrics such as the area under the receiver operating characteristic (ROC) curve (AUC), accuracy, sensitivity, specificity, positive predictive value (PPV), and negative predictive value (NPV). The optimal cutoff value was determined by maximizing the Youden index (sensitivity + specificity − 1). Calibration and decision curves were used to evaluate the model’s calibration and clinical decision-making capability.

### 2.4 Model explanation

The SHAP method quantifies the contribution of each input feature to the final prediction by leveraging concepts from cooperative game theory, addressing the “black-box” nature of ML models (8). This approach incorporates global and local explanations. Global explanations reveal the impact of features on the overall model, whereas local explanations examine the contribution of features in specific samples. The decision-making process of the final model is visually depicted, with both global and local explanations provided via the SHAP method.

### 2.5 Webpage deployment

To facilitate its application in clinical contexts, the final prediction model was integrated and released into a web application built with the Streamlit Python library. When values are entered into the required features settings, the application generates a risk score for VAP in individual IS patients, as well as a force plot showing the effect of each feature on the risk assessment.

### 2.6 Statistical analysis

Data preprocessing, model construction, performance evaluation, and result visualization were conducted using Python (version 3.9.18) and R (version 4.3.2). The variance inflation factor (VIF) was employed to assess potential multicollinearity among the selected features, as detailed in Supplementary Table 3. Continuous variables with a normal distribution were presented as means and standard deviations, while non-normally distributed data were reported as medians (m) and inter-quartile range (IQR). Categorical variables were presented as numbers (*n*) and percentages (%). Differences in continuous variables were compared using Student’s t-tests or Wilcoxon rank-sum tests, while categorical variables were analyzed using Chi-square tests or Fisher’s exact tests. Statistically significant differences were defined as two-tailed *p* values <0.05.

## 3 Results

### 3.1 Patient characteristics

Using ICD-9/10 codes and the defined inclusion–exclusion criteria, we extracted data on 401 and 118 IS patients from versions 2.2 and 3.0 of the MIMIC-IV database, respectively. In the derivation cohort, 149 IS patients developed VAP, while 64 patients in the test cohort experienced VAP. Table 1 outlines the baseline characteristics of both cohorts. The baseline characteristics of the VAP and non-VAP groups in the derivation cohort are detailed in Supplementary Table 4.

**Table 1:** Baseline characteristics of the derivation and test cohorts.

Variables	Derivation cohort	Test cohort	*p-*value
	(*n* = 401)	(*n* = 118)	
Demographics			
Age, years	67.0 (56.0, 77.0)	67.0 (59.3, 72.8)	0.669
Gender, male, *n* (%)	220 (54.9)	73 (61.86)	0.178
Height, cm	168 (160, 178)	170 (163, 178)	0.172
Vital signs			
Heart rate, bpm	85 (74, 96)	85 (74., 98)	0.919
SBP, mmHg	123 (110, 135)	117.50 (107, 128)	0.011
DBP, mmHg	63 (56, 71)	63 (54, 68)	0.461
MBP, mmHg	80 (73, 89)	79 (74, 86.75)	0.674
Respiratory rate, bpm	19 (17, 23)	21 (18, 24)	0.008
Temperature, °C	37.2 (36.8, 37.6)	37.2 (36.8, 37.5)	0.144
SpO2, %	98 (97, 99)	98 (96, 99)	0.044
Laboratory tests			
Hematocrit, %	31.0 (27.5, 36.2)	31.0 (26.6, 35.0)	0.212
Hemoglobin, g/dL	10.3 (9.0, 12.0)	10.0 (8.4, 11.4)	0.018
Platelet, K/μL	188 (131, 258)	202 (142, 276)	0.526
WBC, K/μL	12.0 (9.0, 15.8)	13.1 (9.4, 17.0)	0.202
Aniongap, mmol/L	14 (12, 16)	13 (11, 15)	<0.001
Bicarbonate, mmol/L	22 (20, 25)	23 (20, 26)	0.631
Creatinine, mg/dL	1.1 (0.8, 1.7)	1.3 (0.9, 2.1)	0.014
BUN, mg/dL	21.0 (14.0, 35.0)	27.0 (17.3, 42.8)	0.005
Glucose, mg/dL	143 (117, 182)	162 (131, 195)	0.003
Sodium, mmol/L	140 (137, 143)	140 (136, 144)	0.940
Potassium, mmol/L	4.1 (3.8, 4.5)	4.2 (3.8, 4.6)	0.106
Calcium, mg/dL	8.2 (7.9, 8.7)	8.4 (8.0, 8.8)	0.140
Chloride, mmol/L	106 (102, 110)	105 (99, 108)	0.009
INR	1.3 (1.2, 1.5)	1.3 (1.2, 1.5)	0.409
PT, s	14.1 (12.7, 16.4)	14.1 (12.7, 16.6)	0.747
PTT, s	31.7 (27.6, 40.9)	31.2 (27.8, 44.4)	0.862
PO2, mmHg	143 (106, 201)	108 (85, 144)	<0.001
PCO2, mmHg	38 (34, 44)	42 (36, 46)	0.007
PH	7.4 (7.3, 7.4)	7.4 (7.3, 7.4)	0.041
Base excess, mmol/L	-1 (−3, 1)	-1 (−5, 2)	0.751
A-ado2	198 (139, 284)	225 (156, 303)	0.085
PaO2/FiO2	242 (172, 337)	187 (128, 299)	<0.001
Ventilator settings			
Tidal volume, mL	476.8 ± 88.2	443.4 ± 96.4	<0.001
Minute volume, L/min	9.2 (7.7, 10.5)	8.9 (7.8, 10.0)	0.163
Plateau pressure, cmH_2_O	18 (15, 21)	18 (16, 22)	0.426
PEEP, cmH_2_O	5 (5, 8)	6 (5, 9)	0.210
FiO2, %	50 (44, 58)	50 (40, 60)	0.192
Comorbidities, *n* (%)			
Smoking history	82 (20.5)	28 (23.7)	0.443
Hypertension	191 (47.6)	41 (34.8)	0.013
Diabetes	139 (34.7)	51 (43.2)	0.090
Hyperlipidemia	144 (35.9)	36 (30.5)	0.279
Chronic heart failure	59 (14.7)	26 (22.0)	0.059
Chronic pulmonary disease	59 (14.7)	12 (10.2)	0.207
Chronic liver disease	12 (3.0)	2 (1.7)	0.659
Chronic renal disease	88 (22.0)	32 (27.1)	0.241
Others			
GCS	15 (9, 15)	15 (10, 15)	0.136
Dysphagia, *n* (%)	64 (16.0)	26 (22.0)	0.126
Tracheostomy, *n* (%)	10 (2.5)	6 (5.1)	0.259
Tube feed, *n* (%)	73 (18.2)	30 (25.4)	0.084
LOS before MV, days	0.1 (0.0, 1.4)	0.5 (0.0, 3.2)	0.026
Antibiotic counts	1 (0, 3)	1 (0, 3)	0.586
Suctioning counts	4 (2, 5)	3 (1, 4)	<0.001
Invasive line counts	2 (1, 3)	0 (0, 1)	<0.001

SBP, systolic blood pressure; DBP, Diastolic blood pressure; MBP, Mean blood pressure; A-aDO2, pulmonary alveolus-arterial difference of oxygen pressure; PaO_2_/FiO_2_, the partial pressure of arterial oxygen/ fraction of inspired oxygen; PEEP, positive end-expiratory pressure; GCS, Glasgow Coma Scale; LOS before MV, length of stay before mechanical ventilation.

### 3.2 Selection of features

Six of the initial 59 potential predictive features were removed due to a missing rate of more than 20%, while 7 were excluded due to a correlation coefficient greater than 0.7, as shown in Supplementary Table 5 and Supplementary Figure 2. Additionally, 39 features were removed during the Boruta algorithm phase. After considering clinical practicality, 7 features were eventually included to construct the ML models, including Systolic Blood Pressure (SBP), Diastolic Blood Pressure (DBP), International Normalized Ratio (INR), Length of Stay before Mechanical Ventilation (LOS Before MV), Dysphagia, Antibiotic counts, and Suctioning counts, as illustrated in Figure 2.

### 3.3 Model performance

**Figure 3:**
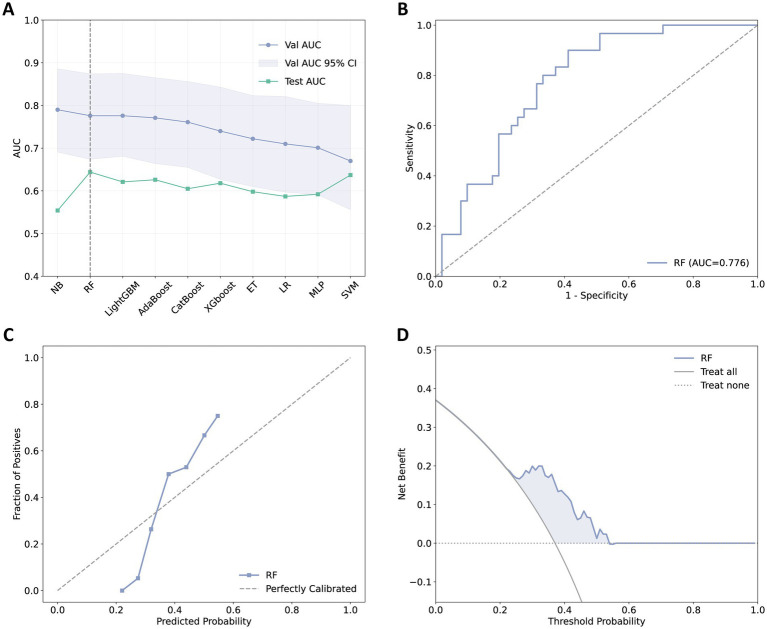
Selection and performance visualization of the final model. **(A)** AUC values for 10 machine learning models in the validation and test sets, with the final selected model indicated by a dashed line. **(B)** ROC curve for the final model. **(C)** Calibration curve for the final model. **(D)** Decision curve for the final model.

The performance of the 10 ML models is presented in Table 2 and Supplementary Table 6. In the derivation cohort, the NB model performed best with an AUC of 0.790, followed by the RF and LightGBM models, both of which had an AUC of 0.776. In the test cohort, the RF model exhibited the best generalization capability with an AUC of 0.644. Figure 3A compares the AUC values of the 10 ML models in the internal validation and external test sets, while Figures 3B–D present the ROC curve, calibration curve, and decision curve of the final model.

**Table 2:** Performance of 10 machine learning models in validation and test cohort.

Models	Val AUC	Accuracy	Sensitivity	Specificity	PPV	NPV	Test AUC
NB	0.790	0.728	0.867	0.647	0.591	0.892	0.554
RF	0.776	0.704	0.900	0.588	0.563	0.909	0.644
LightGBM	0.776	0.753	0.833	0.706	0.625	0.878	0.621
AdaBoost	0.771	0.753	0.800	0.725	0.632	0.860	0.626
CatBoost	0.761	0.716	0.867	0.627	0.578	0.889	0.605
XGboost	0.740	0.741	0.667	0.784	0.645	0.800	0.618
ET	0.722	0.642	0.867	0.510	0.510	0.867	0.598
LR	0.710	0.642	0.767	0.569	0.511	0.806	0.587
MLP	0.701	0.630	0.833	0.510	0.500	0.839	0.592
SVM	0.670	0.630	0.800	0.529	0.500	0.818	0.637

AdaBoost, adaptive boosting; AUC, area under the receiver-operating-characteristic curve; CatBoost, category boosting; ET, extra trees; LightGBM, light gradient boosting machine; LR, logistic regression; MLP, multilayer perceptron; NB, naive bayes; NPV, negative predictive value; PPV, positive predictive value; RF, random forest; SVM, support vector machine; XGboost, eXtreme gradient boosting.

### 3.4 Model explanation

**Figure 4:**
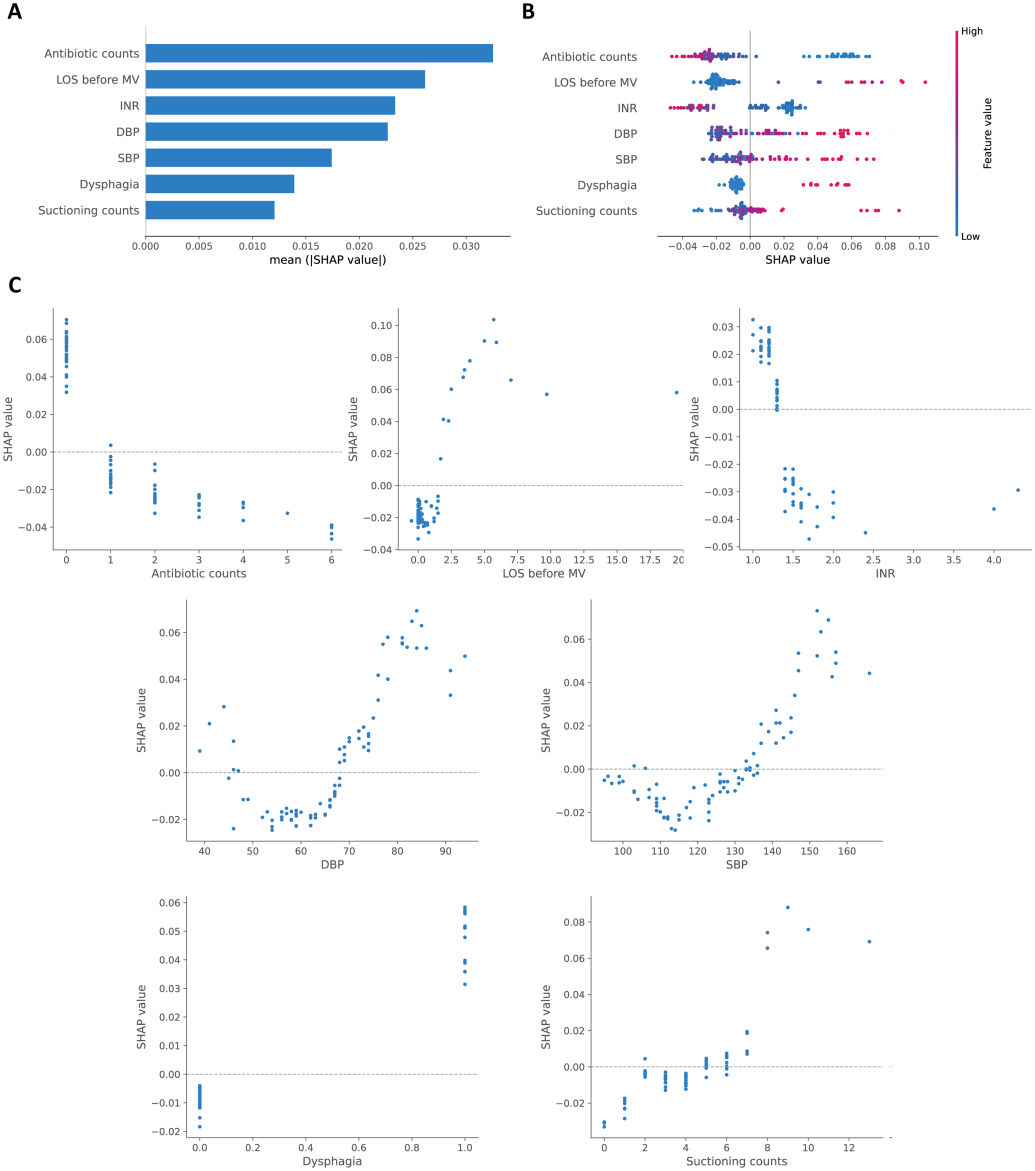
Global SHAP interpretation of the final model. **(A)** Global bar plot of SHAP values. **(B)** Global beeswarm plot of SHAP values. **(C)** Global dependence plots for individual features.

The SHAP method was employed to interpret the final model. Figure 4A presents a bar plot of features ranked by their mean absolute SHAP values. Figure 4B shows a beeswarm plot, illustrating the relationship between each feature’s value and the predicted risk of VAP. These plots indicate that antibiotic counts, LOS before MV, and INR are the top three contributors. Figure 4C shows SHAP dependence plots that further reveal the distribution of each feature and its global relationship with VAP risk.

**Figure 5:**
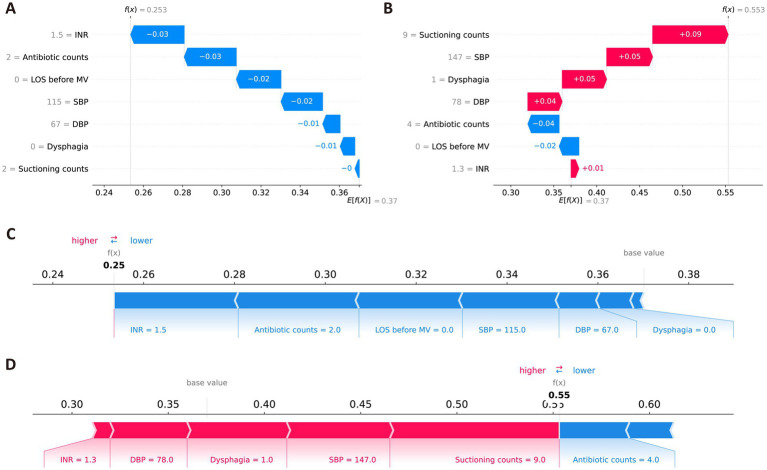
Local SHAP interpretations of the final model. **(A,C)** Representative waterfall and force plots for ischemic stroke patients without VAP. **(B,D)** Representative waterfall force plots for ischemic stroke patients with VAP. Red indicates that the feature positively contributes to the risk of VAP; blue indicates a negative contribution.

In addition, the SHAP method was used to conduct local interpretation for the final model. Figure 5 shows detailed local interpretations using SHAP waterfall plots and force plots. As shown in Figure 5A, for patient who did not eventually develop VAP, the SHAP analysis indicates that higher INR, the administration of antibiotics twice, a shorter LOS before MV, normal SBP and DBP, the absence of dysphagia, and fewer suctioning operations negatively contribute to the model’s prediction of VAP, resulting in a low-risk classification. In contrast, as depicted in Figure 5B, for patient who eventually developed VAP, the SHAP analysis reveals that multiple suctioning operations, higher SBP, and the presence of dysphagia positively support the prediction of VAP, while the administration of antibiotics four times and a shorter LOS before MV have a negative impact. The aggregated contributions of these features incline the model toward predicting a high risk of VAP for the patient.

### 3.5 Online application

**Figure 6:**
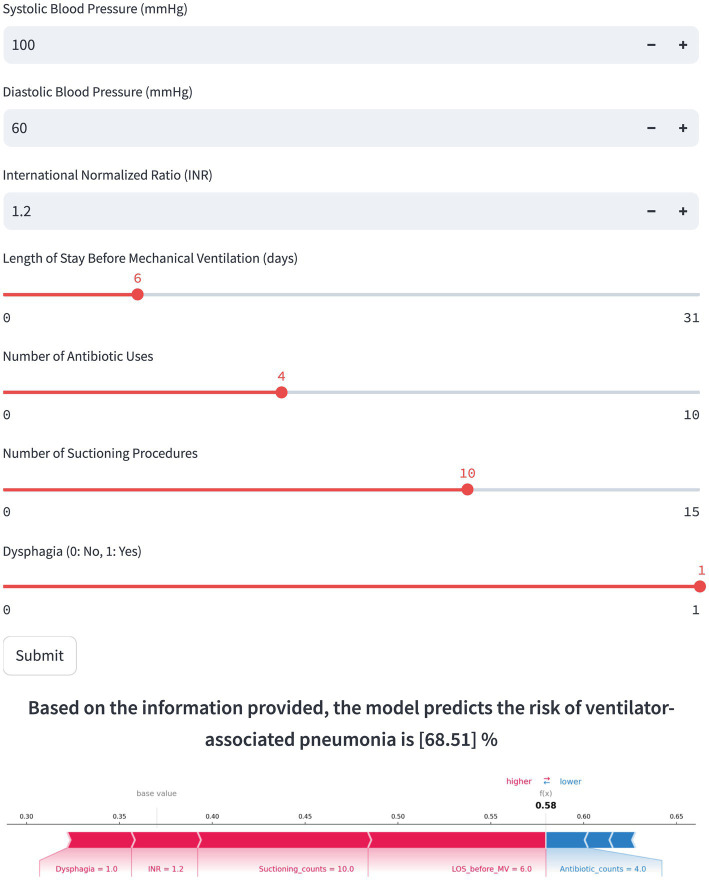
The web application deployment based on the final model.

Based on the final RF model, an interactive web-based tool was developed to facilitate clinical application.^1^ Clinicians can input patient-specific clinical parameters to obtain an individualized prediction of VAP risk, along with a SHAP force plot clearly depicting each feature’s contribution. As illustrated in Figure 6, red features on the left side like LOS Before MV, suctioning counts, INR, and dysphagia push the prediction toward “VAP,” while the blue features on the right side like antibiotic counts, DBP, and SBP drive the prediction toward “non-VAP.” For the selected scenario shown in Figure 6, the model predicts a 68.51% probability of VAP occurrence, indicating a high risk for VAP.

## 4 Discussion

This study developed and validated a ML model using clinical features to predict the risk of VAP in IS patients in the ICU, based on an open-source database. We employed the Boruta algorithm for feature selection before building the model, which enabled the identification of seven predictive parameters and the use of a limited number of clinical variables to enhance clinical practicality. SBP, DBP, INR, suctioning and antibiotic counts were extracted within the first 24 h of MV, allowing for a short predictive window to identify IS patients at high risk of developing VAP. To the best of our knowledge, this study is the first to predict VAP risk in ICU IS patients using an interpretable ML approach.

Our study found dysphagia as a significant risk factor for the incidence of VAP in stroke patients, which is consistent with prior research (13, 14). Stroke patients often experience dysphagia, with incidence rates ranging from 30 to 50% (15, 16). Dysphagia is associated with prolonged hospital stays, increased healthcare costs, and an elevated risk of persistent disability and mortality (17–21). Due to impaired swallowing function and a diminished cough reflex, patients with dysphagia struggle to clear oral secretions, rendering them more susceptible to aspiration events and subsequent pneumonia. Recent studies have further confirmed that stroke-related dysphagia significantly increases the risk of pulmonary infection, underscoring the clinical importance of early dysphagia screening in stroke patients (22, 23). Large-scale prospective studies have reported similar findings, demonstrating that dysphagia following acute ischemic stroke markedly increases the risk of pneumonia and is independently associated with poor outcomes and higher mortality (24, 25). Furthermore, early dysphagia screening (within 24 h of admission) has been proven to reduce the risk of stroke-associated pneumonia (24).

Suctioning of secretions, primarily subglottic secretion drainage, is a commonly recommended treatment for clearing lower airway secretions using an endotracheal tube to prevent VAP. Multiple studies have demonstrated that subglottic suctioning can significantly reduce the incidence of VAP by limiting microbial colonization around the endotracheal tube (26, 27). However, our study observed a significant link between increased suctioning frequency and a higher risk of VAP in IS patients, which is consistent with the findings of Abdallah et al. (28). Although this may seem contradictory, frequent invasive suctioning can impair mucociliary function and compromise airway mucosal integrity, thereby weakening the natural immune barrier and increasing susceptibility to bacterial colonization (29, 30). Moreover, excessive suctioning may introduce exogenous pathogens into the respiratory tract, further elevating the risk of infection (30). A higher frequency of suctioning may also reflect a greater secretion burden, suggesting the presence of a subclinical or early-stage infection. Our findings highlight the importance of carefully balancing suctioning frequency while effectively managing airway secretions to minimize harm, especially in vulnerable populations such as IS patients.

INR, a critical indicator of coagulation function, was also identified as a significant risk factor for VAP in IS patients. In the context of a stroke, an increased INR usually indicates the use of vitamin K antagonists, especially warfarin. Warfarin has been shown to have a protective effect for community-acquired pneumonia, probably due to its effect on disturbed thrombin formation and alveolar fibrin deposition (31). Our findings revealed that IS patients with higher INR had a lower risk of VAP, which may be due to warfarin medication. The underlying mechanism deserves further investigation in larger populations.

According to current guidelines on VAP treatment (32), prophylactic antibiotic use is generally not recommended to prevent VAP, due to the risk of long-term or unnecessary antibiotic use fostering resistant bacterial strains, increasing the burden of antibiotic resistance for both patients and hospitals. However, our findings suggest that administering antibiotics within the first 24 h of MV may lower the incidence of VAP. We speculate that this effect may be attributed to the serious and intricate nature of stroke as well as the existence of concomitant infectious diseases in the ICU. An early and appropriate use of antibiotic may reduce the incidence of VAP by preventing the colonization and proliferation of potential pathogens in the respiratory tract.

Our research indicates that a longer ICU stay before MV may increase the incidence of VAP, which is consistent with previous studies (33, 34), presumably due to the positive link between ICU stay duration and infection risk. Furthermore, the SHAP dependency plots in this study suggest a potential non-linear relationship between DBP, SBP, and the risk of VAP. Higher values of DBP and SBP are associated with an increased risk of VAP, while in the lower blood pressure range, although there is a tendency for low blood pressure to elevate the risk of VAP, the limited number of data points prevents us from drawing a definitive conclusion. Future studies should incorporate more data from patients with low blood pressure to further investigate and clarify this potential non-linear relationship.

ML excels in processing and analyzing complex multimodal and high-dimensional data. However, ML algorithms’ complicated nature makes it difficult to understand how they make prediction and decisions, presenting a “black box” issue that hinders its widely use in healthcare. As highlighted by Stinear et al. (35), developing operational and interpretable ML models is crucial for clinical practice. In this study, we utilized the SHAP method to address the “black box” problem of ML models. SHAP, a unified framework for ML interpretability proposed by Lundberg et al. (8), quantifies the contribution of each feature in the model to the final prediction, aiming to enhance user understanding of decision-making processes and increase confidence and trust in the predictive model’s outcomes. In addition, we deployed a web-based application that medical staff can use to predict VAP in IS patients, and we released it to the public based on the final RF model.

This study has several limitations. Firstly, it is a single-center retrospective study, utilizing health data from multiple time periods at this center for model training, validation, and testing. External validation at additional medical centers is necessary to further evaluate the model’s generalizability. Relying on a single database may also introduce potential data quality issues and selection bias. Secondly, identifying VAP patients using ICD codes in the MIMIC-IV database presents challenges in retrospectively determining the exact timing of VAP diagnosis. Lastly, our study focused primarily on the average or cumulative occurrence of clinical features within the first 24 h of MV, ignoring the impact of dynamic changes in clinical features during ICU stay. Therefore, future multicenter prospective studies conducted across diverse clinical settings are needed to comprehensively validate the robustness and applicability of our predictive model.

## 5 Conclusion

We developed and evaluated multiple ML algorithms to determine the risk of VAP in ICU patients with IS. Both the internal validation and the external testing showed that the RF model performed reliably. Our findings indicate that SBP, DBP, INR, antibiotic usage, frequency of suctioning within the first 24 h of MV, LOS Before MV, and dysphagia have a substantial impact on risk assessment. The ML model and online web tool developed in this study can help clinicians identify high-risk IS patients for VAP effectively at an early stage. Further multicenter prospective studies are warranted to validate the model’s generalizability and practical utility.

## Data Availability

The datasets presented in this study can be found in online repositories. The names of the repository/repositories and accession number(s) can be found in the article/Supplementary material.

## Footnotes

^1^ https://isvaprisk.streamlit.app/
